# The Characteristic of S100A7 Induction by the Hippo-YAP Pathway in Cervical and Glossopharyngeal Squamous Cell Carcinoma

**DOI:** 10.1371/journal.pone.0167080

**Published:** 2016-12-01

**Authors:** Fei Kong, Yunguang Li, Enze Hu, Rui Wang, Junhao Wang, Jin Liu, Jinsan Zhang, Dacheng He, Xueyuan Xiao

**Affiliations:** 1 Key laboratory of Cell Proliferation and Regulation Biology, Ministry of Education, Beijing Normal University, Beijing, China; 2 School of Pharmaceutical Sciences and Key Laboratory of Biotechnology and Pharmaceutical Engineering, Wenzhou Medical University, Wenzhou, Zhejiang, China; Toho Daigaku, JAPAN

## Abstract

S100A7 is expressed in many squamous cell carcinomas (SCCs). Our previous study revealed that S100A7 was dramatically induced in several SCC cells and activation of the Hippo pathway significantly promoted S100A7 in epidermoid carcinoma cells. However, whether the Hippo pathway regulates S100A7 expression in SCCs remains largely unknown. Here, we uncover that S100A7 induction by the Hippo-YAP pathway displays different characteristic in cervical and glossopharyngeal SCC. In well differentiated HCC94 cervical cells and FaDu pharyngeal cells, S100A7 is easily induced by both suspension and dense culture, which is accompanied by an increase in YAP phosphorylation and a decrease in nuclear YAP. Strikingly, these correlations of S100A7 and YAP reverse after recovery of cell attachment or relief from dense culture. Further examination finds that S100A7 induction is significantly repressed by nuclear YAP, which is validated by activation or inhibition of the Hippo pathway via loss- and/or gain-of- LATS1 and MST1 function. Subsequently, we prove that TEAD1 is required for YAP transcriptional repression of S100A7. However, S100A7 is hardly induced in poorly differentiated SiHa cervical cells and NCI-H226 pulmonary cells even in suspension or activation of the Hippo pathway. More importantly, cervical and lingual SCC tissues array analyses show that S100A7 expression displays the positive correlation with pYAP-S127 and the negative correlation with nuclear YAP in the majority of well differentiated but not in poorly differentiated tissues. Collectively, our findings demonstrate that the different induction of S100A7 toward activation of the Hippo pathway mainly depends on the degree of cell differentiation in cervical and glossopharyngeal SCC.

## Introduction

Squamous cell carcinomas (SCCs) are the most common cancer and can be very aggressive and metastatic. S100A7 (psoriasin) belongs to the S100 multigenic family of calcium-modulated proteins of the EF-hand type and is originally identified in psoriatic keratinocytes.[1,2] Subsequent studies have shown that upregulation of S100A7 is detected in nearly all types of SCC tissues as well as adenocarcinomas of the breast.[3–10] Our previous study indicated that S100A7 expression can be significantly induced depending on the cell density and cell morphology in several SCC cells and xenografts.[11,12] Recently, we have uncovered that activation of the Hippo pathway significantly promote S100A7 expression in epidermoid carcinoma A431 cells.[13] However, little is known whether the Hippo pathway is involved in S100A7 induction in SCCs. Therefore, understanding the mechanisms and characters of S100A7 induction in these SCCs has significant implications for elucidating the mechanism of SCCs development and treatment.

The Hippo pathway is a newly established tumor suppressor pathway that plays a central role in tissue homoeostasis.[14] At the core of this pathway in mammals is a kinase cascade consisting of MST1/2 and LATS1/2. MST1/2 phosphorylates the hydrophobic motif of LATS1/2 (LATS-HM) and activates the LATS1/2,[15] which in turn directly phosphorylates YAP (Yes-associated protein) at Serine 127 (YAP-S127).[15–19] The phosphorylation of YAP-S127 is required for its cytoplasmic retention, wherein it can no longer acts as a transcriptional coactivator and also not promotes or represses YAP-dependent gene expression via binding with TEAD (TEA domain) as YAP in nucleus.[19] Recent studies demonstrate a requirement for the Hippo-YAP pathway to sense the cues from cell morphology and cell density via actin cytoskeleton reorganization.[20,21]

Here we report that S100A7 is inducible in well differentiated HCC94 and FaDu SCC cells but not in poorly differentiated H226 and SiHa cells. We further demonstrate that S100A7 induction in HCC94 and FaDu SCC cells is repressed by YAP/TEAD1 via activation of the Hippo pathway. The negative correlation of S100A7 expression and nuclear YAP is detected in well differentiated cervical and glosspharyngeal SCC cells and tissues. Thus, our findings provide a new insight for understanding the characteristic of S100A7 induction by the Hippo-YAP pathway in cervical and glossopharyngeal SCC.

## Materials and Methods

A full description of materials and methods, including Plasmids and Reagents, Western blot, Immunofluorescence staining, Immunohistochemistry, MTT assay and Statistical analysis was described in S1 Text.

### Cell culture

Human squamous carcinoma cell lines HCC94, FaDu, SiHa and NCI-H226 were purchased from the Chinese Academy of Sciences Committee Type Culture Collection Cell Bank and were authenticated by short tandem repeat analysis at HK Gene Science Technology Co. (Beijing, China). All cells were cultured according to the corresponding culture methods of the ATCC and Chinese Academy of Sciences Committee Type Culture Collection Cell Bank. Cell suspension cultures were obtained as described in our previous studies.[11] Cultures with different cell densities were achieved by plating cells at low cell density (here-after called ‘sparse’, 7 500 cells/cm^2^) and at high cell density (‘dense’, 75 000–100 000 cells/ cm^2^).

### siRNA and transfection

To silence the expression of YAP, LATS1, MST1, TEAD1, TEAD2, TEAD3 and TEAD4, all siRNAs as well as the non-targeting control siRNA were purchased from Gene Pharma (Shanghai, China) and transfected using the Transfection Reagent (Polyplus, NY, USA) according to the manufacturer's protocol. For each gene, two individual siRNAs were used (S1 Table).

### Reverse transcription and quantitative RT-PCR

Total RNA was extracted from cells for the generation of single-stranded cDNA. Quantitative RT-PCR (qPCR) was performed using an ABI 7300 Real-time PCR System (Life Technologies Ltd, Paisley, UK) with the Power SYBR® Green PCR Master Mix (Life Technologies Ltd) in a final volume of 20 μL. GAPDH was used as an endogenous control for each sample. The primers used for each of the genes are listed (S2 Table).

### Tissue specimens

Lingual SCC (No. OR601b) and uterine cervix SCC (No. CR803) tissue microarrays were purchased from Xi’ an Alenabio Company (Xian, China). All patients with cancer had received a pathological diagnosis and none had received prior therapy. All cancer tissues were obtained from surgically treated patients who gave their written informed consent to participate in this study and the Ethics Committee of the General Hospital of PLA Rocket Force (No. KY2015031) approved this protocol.

## Results

### Identical activation of the Hippo pathway but different induction of S100A7 in cervical and pharyngeal SCC cells

**Fig 1:**
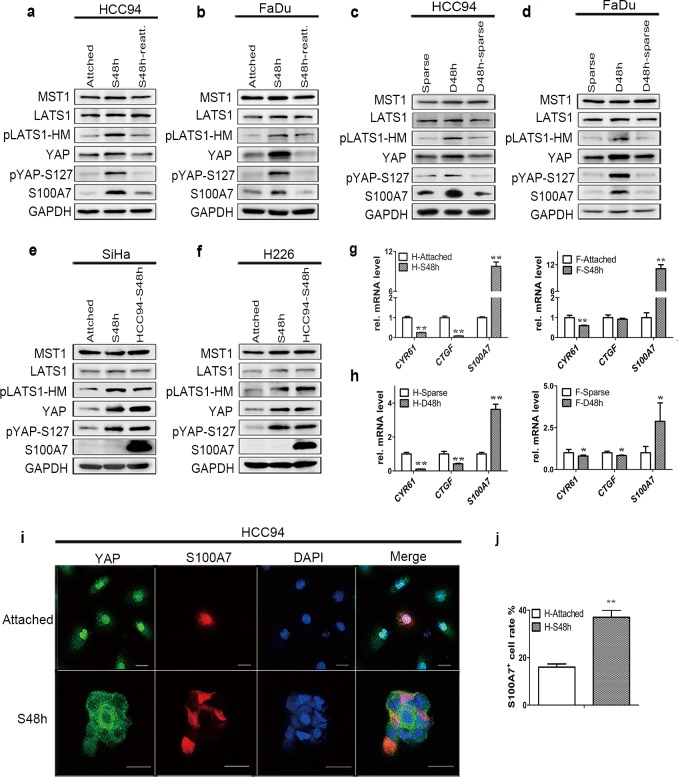
**Identical activation of the Hippo pathway but different induction of S100A7 in cervical and pharyngeal SCC cells** (a, b) Suspension culture induced the expression of S100A7, pYAP-S127 and pLATS1-HM is detected by western blot in HCC94 (a) and FaDu (b) cells. Cells were cultured in suspension for two days (S48h) and then reattachment for one day (S48h-reatt.). (c, d) Western blots showing dense culture induced the expression of S100A7, pYAP-S127 and pLATS1-HM in HCC94 (c) and FaDu (d) cells. Cells were cultured densely for two days (D48h) and then relief from dense culture (D48h-sparse). (e, f) Suspension culture induced the expression of S100A7, pYAP-S127 and pLATS1-HM is detected by western blot in SiHa (e) and H226 (f) cells. HCC94-S48h was used as the positive control. GAPDH was used as a loading control. (g, h) The mRNA levels of *CYR61*, *CTGF* and *S100A7* are analyzed by qRT-PCR in HCC94 and FaDu cells. H: HCC94 cells; F: FaDu cells. Error bar, SD of three different experiments. *P<0.*05*, **P<0.01; *t-test*. (i) Representative immunofluorescence images of S100A7 and YAP subcellular location is detected in attached and suspended HCC94 cells. DAPI is a nuclear counterstain. Scale bar, 20μm. (j) Quantification of percentage of the S100A7 positive HCC94 cells was performed in three randomly chosen fields. Typically, each field contains 40–60 cells and are all counted.

Our recent data show YAP as a suppressor in S100A7 induction in epidermoid carcinoma cells.[13] Since the upregulation of S100A7 displayed the positive correlation with the degree of differentiation in clinical SCC tissues;[22] we selected three cervical and pharyngeal SCC cell lines with the different degrees of differentiation in order to investigate the role of YAP in S100A7 induction. These cells were cultured in suspension or at two different cell densities, including sparse and dense (S1 Fig). Interestingly, we found that S100A7 was easily induced in well differentiated HCC94 and FaDu cells; which were accompanied by an increase in YAP-S127 phosphorylation in suspended cells compared with attached cells (Fig 1A and 1B). Similar phenomena also occurred in dense cells compared with sparse cells (Fig 1C and 1D). Strikingly, these correlations of S100A7 and YAP reverse after recovery of cell attachment or relief from dense culture. However, in poorly differentiated SiHa cells (Fig 1E), the expression of S100A7 was very low and was hardly induced although pYAP-S127 was also significantly increased in suspended cells. Similar results were also obtained in poorly differentiated H226 pulmonary SCC cells (Fig 1F). Considering YAP as the effector of the Hippo pathway, we further examined the expression of other Hippo pathway core components, including MST1 and LATS1 in these cells. Consistently, the Hippo pathway was activated in all tested SCC cells by suspension and/or dense culture, as indicated by the status of LATS-HM (LATS1-T1079) phosphorylation (Fig 1A–1F). To further confirm the activation of the Hippo pathway, *CTGF* (Connective tissue growth factor) and *CYR61* (Cysteine-rich angiogenic inducer 61), two direct endogenous markers of YAP, were analyzed by quantitative RT-PCR (qPCR) as readout of YAP activity. Consistent with the increased YAP phosphorylation, *CTGF* and/or *CYR61* transcription are significantly suppressed in suspended and dense HCC94 and FaDu cells (Fig 1G and 1H). Similar results were also obtained in suspended H226 and SiHa cells (data not shown). Using immunofluorescence, we further examined the expression pattern of S100A7 and YAP in HCC94 cells. Representative immunofluorescence images are shown in Fig 1I. In line with these finding, YAP markedly translocated to the cytoplasm in suspended cells and the percentage of S100A7-positive cells was significantly increased from 16% to 37% (Fig 1J). Collectively, our data uncover the characteristic of S100A7 induction and the correlation between S100A7 and YAP in cervical and pharyngeal SCC cells.

### Activation of the Hippo pathway promoted S100A7 induction in well differentiated cervical and pharyngeal SCC cells

**Fig 2:**
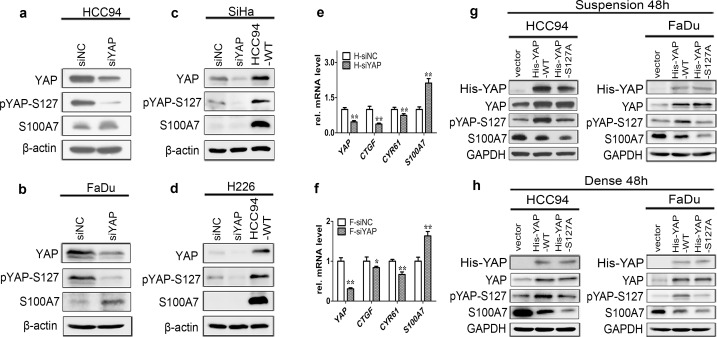
**The nuclear YAP is responsible for inhibition of S100A7 expression in well differentiated cervical and pharyngeal SCC cells** (a-d) Depletion of YAP using siRNA in normal attached HCC94 (a), FaDu (b), SiHa (c) and H226 (d) cells. The expression of YAP, S100A7 and pYAP-S127 is determined by western blotting. HCC94-WT was used as the positive control. β-actin was used as a loading control. (e, f) qRT-PCR analyses of *YAP*, *S100A7*, *CTGF* and *CYR61* in attached HCC94 (e) and FaDu (f) cells after silencing of YAP. H: HCC94 cells; F: FaDu cells. Error bar, SD of three different experiments. *P<0.*05*, **P<0.01; *t-test*. (g, h) HCC94 and FaDu cells were transfected with YAP-WT and mutant activated YAP-S127A. Subsequently, the cells were cultured in suspension (g) or dense (h) for two days. The expression of S100A7, YAP and pYAP-S127 is detected by western blotting. Anti-His tag antibody was used to judge the transfection efficiency. GAPDH was used as a loading control.

**Fig 3:**
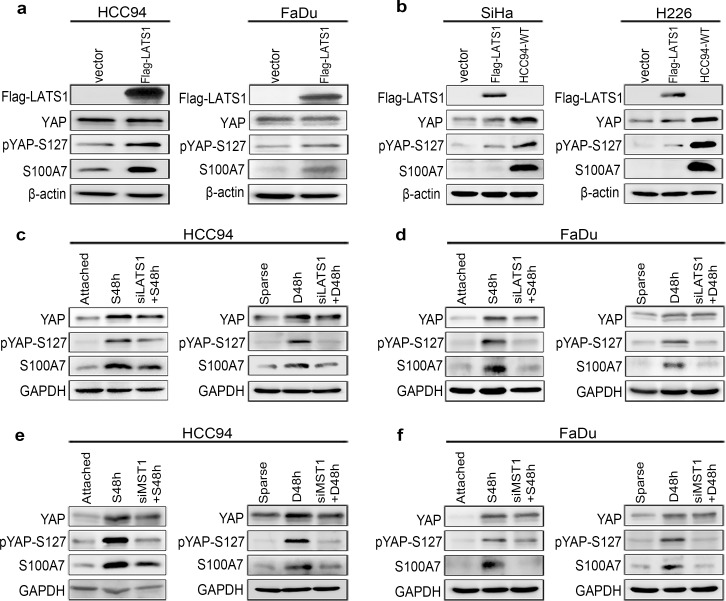
The Hippo pathway is responsible for S100A7 induction in well differentiated cervical and pharyngeal SCC cells. (a, b) Overexpression of LATS1 in normal attached HCC94 (a, left), FaDu (a, right), SiHa (b, left) and H226 (b, right) cells. Anti-Flag tag antibody was used to judge the transfection efficiency. HCC94-WT was used as the positive control. β-actin was used as a loading control (c, d) Depletion of LATS1 using siRNA attenuates S100A7 in suspended or dense HCC94 (c) and FaDu (d) cells. siLATS1+S48h (or D48h) indicates that cells are cultured in suspension (or dense) for 48 h after silencing of LATS1. (e, f) Silencing MST1 in HCC94 (e) and FaDu (f) cells cultured in suspension or dense. siMST1+S48h (or D48h) indicates that cells are cultured in suspension (or dense) for 48 h after losing of MST1. GAPDH was used as a loading control.

We next sought to explore whether the Hippo-YAP is really involved in S100A7 induction in cervical and pharyngeal SCC cells. We silenced the expression of YAP in attached SCC cells in order to mimic cells grown in suspension or dense condition. We found that single depletion of YAP was sufficient to induce S100A7 expression in well differentiated HCC94 and FaDu cells (Fig 2A and 2B) but not in poorly differentiated SiHa (Fig 2C) and H226 cells (Fig 2D). The efficiency of YAP knockdown was also confirmed by a decrease of *CTGF* and *CYR61* expression in HCC94 (Fig 2E) and FaDu (Fig 2F) cells. In contrast, overexpression of YAP-S127A (a constitutively activated form of YAP) repressed suspension- and dense-induced S100A7 expression more effective than YAP-WT in HCC94 and FaDu cells (Fig 2G and 2H). These data collectively support that inhibition of YAP transcriptional activity is indispensable for S100A7 induction in well differentiated cervical and pharyngeal SCC cells. Consistently, activation of the Hippo pathway by overexpression of LATS1 dramatically increased S100A7 expression and YAP phosphorylation in HCC94 and FaDu cells (Fig 3A) but not in SiHa and H226 cells (Fig 3B). Importantly, the opposite results were obtained by silencing of LATS1 and MST1 (S2 Fig) in suspended- and dense- HCC94 and FaDu cells both in protein (Fig 3C–3F) and mRNA levels (S3 Fig). Together, our data unequivocally demonstrate for the first time that activation of the Hippo pathway is the necessary condition for S100A7 induction in well differentiated cervical and pharyngeal SCC cells.

### F-actin disruption enhances S100A7 expression via activation of the Hippo pathway in well differentiated cervical and pharyngeal SCC cells

**Fig 4:**
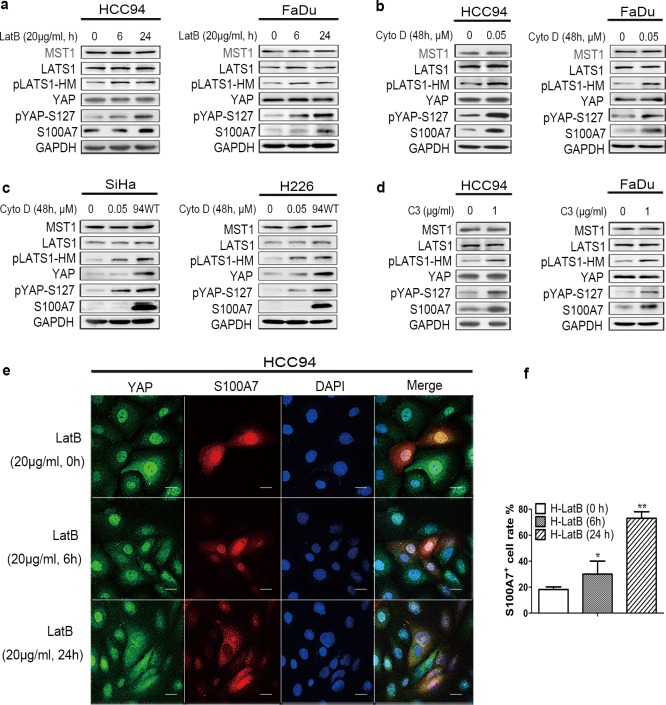
**F-actin disruption induces S100A7 via the activation of the Hippo pathway in well differentiated cervical and pharyngeal SCC cells** (a) HCC94 and FaDu cells were treated with LatB (20 μg/ml) for 6 h and 24 h. (b, c) HCC94 (b, left), FaDu (b, right), SiHa (c, left) and H226 (c, right) cells were cultured for 48 h in the presence of Cyto D (0.05μM). 94WT was used as the positive control. GAPDH was used as a loading control. (d) C3 (1 μg/ml) was added to HCC94 and FaDu cells with serum-free growth medium for 4 h prior to harvesting for western blotting analyses. GAPDH was used as a loading control. (e) Representative immunofluorescence images of S100A7 and YAP subcellular location is detected in HCC94 cells treated with LatB (20 μg/ml) for different time. DAPI is a nuclear counterstain. Scale bar, 20μm. (f) Quantification of percentage of the S100A7 positive HCC94 cells in the presence of LatB was performed in three randomly chosen fields. Typically, each field contains 40–50 cells and are all counted. H: HCC94 cells.

Cell suspension and dense culture leads to activation of the Hippo pathway through actin cytoskeleton remodeling.[20,23] To test whether F-actin cytoskeleton reorganization participates in S100A7 induction through the Hippo-YAP pathway in cervical and pharyngeal SCC cells, two F-actin cytoskeleton-disrupting reagents, Latrunculin B (Lat B) (Fig 4A) and cytochalasin D (Cyto D) (Fig 4B and 4C) were used to treat attached cells. Lat B and Cyto D disrupt the F-actin cytoskeleton by preventing actin polymerization and by capping filament plus ends, respectively.[20] We found that abrogation of the F-actin polymerization by these drugs both resulted in activation of the Hippo pathway in HCC94 and FaDu cells. Lat B and CytoD treated these two well differentiated SCC cells show obviously induction of S100A7 expression at 24h (Fig 4A) and 0.05μM (Fig 4B), respectively. Real-time PCR data further confirmed the decrease of YAP transcriptional activity (S4 Fig). As expected, no similar effect of Cyto D on S100A7 induction was detected in poorly differentiated SiHa and H226 cells (Fig 4C). It has been reported that Rho can strongly induces YAP dephosphorylation via regulation of the actin cytoskeleton.[20] Thus, we asked whether inhibition of Rho performed the similar effect as Lat B and Cyto D on S100A7 induction. Indeed, inhibition of Rho with C3, a specific inhibitor of Rho, not only promoted S100A7 expression and YAP phosphorylation (Fig 4D and S4 Fig), but also activated the Hippo pathway. By immunofluorescence, we observed that disruption of F-actin by Lat B impaired YAP-nuclear translocation and significantly increased the percentage of S100A7-positive cells in HCC94 cells (Fig 4E and 4F). Representative immunofluorescence images are shown in Fig 4E. These results provide the direct evidence to support that F-actin controls S100A7 expression via the Hippo-YAP pathway in well differentiated cervical and pharyngeal SCC cells.

### Repression of S100A7 by nuclear YAP requires TEAD1 in well differentiated cervical and pharyngeal SCC cells

**Fig 5:**
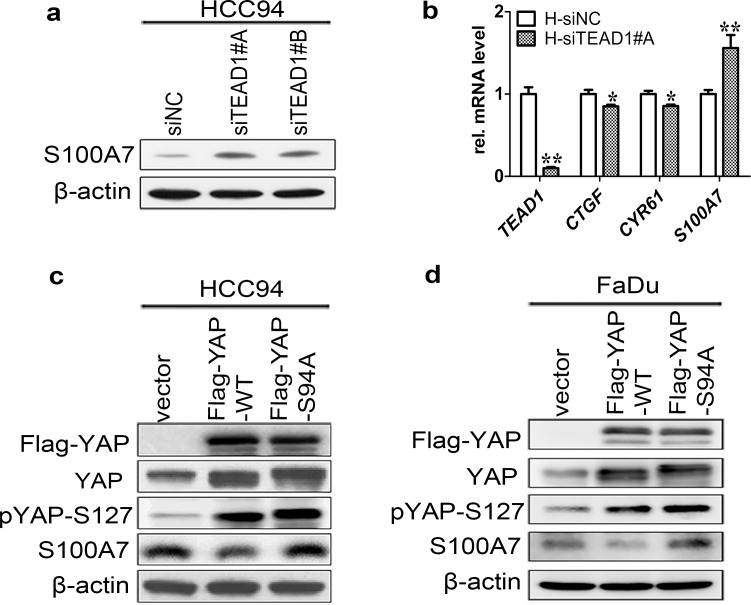
TEAD1 mediates YAP-dependent S100A7 expression. (a) Western blot analyses for S100A7 expression after TEAD1 silencing in normal attached HCC94 cells. (b) The mRNA expression of *TEAD1*, *CTGF*, *CYR61* and *S100A7* is examined using qRT-PCR. H: HCC94 cells. Error bar, SD of three different experiments. *P<0.*05*, **P<0.01; *t-test*. (c, d) Overexpression of YAP-WT and mutant activated YAP-S94A in normal attached HCC94 (c) and FaDu (d) cells. Anti-Flag tag antibody was used to judge the transfection efficiency. β-actin was used as a loading control.

Since YAP did not have DNA-binding activity and TEAD has emerged as one of the main partners of YAP on DNA to stimulate or repress YAP-dependent gene expression,[24,25] we hypothesized that the TEAD may be the key regulator for S100A7 induction. To test this hypothesis, we transiently knocked down the expression of each isoform of TEADs in attached HCC94 using two specific TEADs siRNAs. Importantly, depletion of TEAD1 significantly induced S100A7 expression in the cells (Fig 5A). This effect was specific for TEAD1 only, since silencing of other members of the same family did not have the similar function (S5 Fig). Consistently, a decrease in the expression of *CTGF* and an increase in the expression of S100A7 were confirmed using qPCR (Fig 5B). To further confirm the role of interaction of TEAD1 and YAP on S100A7 induction, we overexpressed YAP-S94A in attached cells because YAP-S94A was defective in TEAD activation. Indeed, S100A7 expression was marginally affected by YAP-S94A compared with the overexpression of YAP-WT (Fig 5C and 5D), indicating that through interaction with TEAD1, nuclear YAP regulates S100A7 expression. Taken together, we conclude that TEAD1 is truly required for the inhibitory effect of YAP on S100A7 induction in well differentiated cervical and pharyngeal SCC cells.

### S100A7 positively correlates with pYAP-S127 and negatively correlates with nuclear YAP in well differentiated cervical and lingual SCC tissues

**Fig 6:**
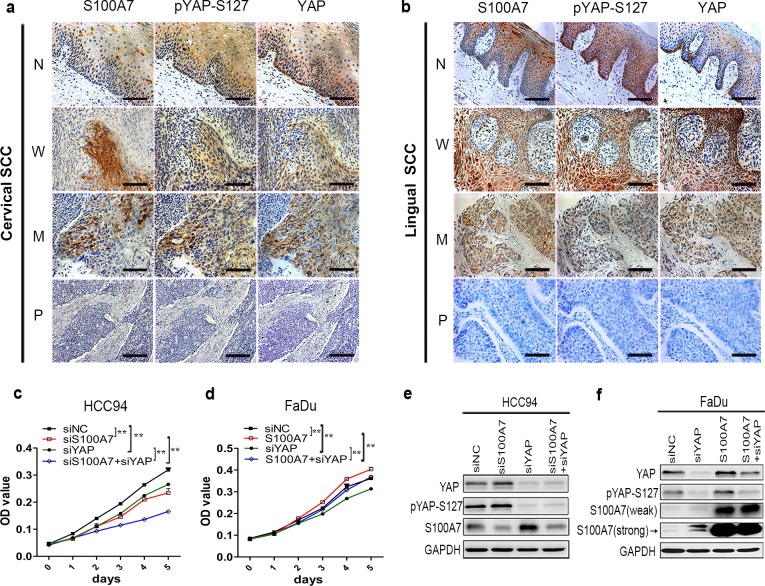
**S100A7 positively correlates with pYAP-S127 and negatively correlates with nuclear YAP in well differentiated cervical and lingual SCC tissues** (a, b) Representative images showing S100A7, pYAP-S127 and total YAP staining in well-(W), moderately-(M), poorly-(P) differentiated uterine cervical SCC (a) and lingual SCC (b). The corresponding adjacent normal tissues (N) were also stained. Scale bar, 100μm. (c) The cell proliferation of single deletion of YAP or S100A7 or both in HCC94 was determined by MTT assay. (d)The cell proliferation of overexpressed of S100A7 or silenced YAP or combined in FaDu cells was determined by MTT assay. Error bar, SD of three different experiments. *P<0.*05*, **P<0.01; *t-test*. (e, f) The transfection efficiency of YAP and S100A7 in HCC94 (e) or FaDu (f) cells was determined by western blot. GAPDH was used as a loading control.

Thus, to further investigate the correlation between S100A7 and YAP in clinical SCC tissues, three consecutive sections of uterine cervical and lingual SCC tissues microarrays were stained by specific S100A7, pYAP-S127 and total YAP antibodies, respectively. Representative immunohistochemistry images are shown in Fig 6A and 6B while all the images of well differentiated cervical SCC tissues are showed in S6 Fig. Notably, the majority of well differentiated and more than half of moderately differentiated SCC specimens showed strong or medium S100A7 staining, whereas all of poorly differentiated SCC tissues did not. Consistently, S100A7 positively correlated with pYAP-S127 and negatively correlated with nuclear YAP only in well- and moderately differentiated cervical and lingual SCC specimens (Fig 6A and 6B and Table 1). These data indicate that S100A7 expression toward the Hippo pathway activation largely depend on the differentiated status of cervical and lingual SCC specimens.

**Table 1:** Correlation of S100A7, pYAP-S127 and YAP in relation to the differentiated status of cervical and lingual SCC specimens.

Group	Differentiation	n	S100A7+	pYAP-S127+	S100A7+/pYAP-S127+^a^	YAP		
						C	C/N^b^	none
Cervical SCC specimens	Well	25	25(100%)	17(68%)	17(68%)	13(52%)	9(36%)	3(12%)
	Moderate	26	16(61.5%)	13(50%)	13(50%)	7(26.9%)	15(57.7%)	4(15.3%)
	Poor	18	0(0%)	4(22%)	0(0%)	2(11.1%)	7(38.9%)	9(50%)
Lingual SCC specimens	Well	42	42(100%)	39(92.9%)	39(92.9%)	15(35.7%)	24(57.1%)	3(7.1%)
	Moderate	3	2(66.7%)	1(33.3%)	1(33.3%)	1(33.3%)	1(33.3%)	1(33.3%)
	Poor	3	0(0%)	0(0%)	0(0%)	0(0%)	0(0%)	0(0%)

a, S100A7+/pYAP-S127+, the number (proportion) of both S100A7 and pYAP-S127 positive tissues

b, C/N, the number (proportion) of YAP expressed both in cytoplasm and nuclear

With integrative results of the cervical and pharyngeal SCC cells as well as the cervical and lingual clinical specimens, we puzzled why S100A7 negatively correlated with nuclear YAP in well differentiated cervical and glossopharyngeal SCC cells and tissues since both YAP and S100A7 were reported to promote cells proliferation,[11,12,16] In an effort to elucidate this doubt, we selected HCC94 and FaDu SCC cells and altered the expression of S100A7 or YAP or both according to their endogenous expression levels. Strikingly, double silencing of YAP and S100A7 suppressed HCC94 cells proliferation more effective than each single deletion (Fig 6C). Similarly, the proliferation of FaDu cells was significantly promoted by overexpression of S100A7 but inhibited by depletion of YAP, and slightly influenced by both combinations (Fig 6D). Silencing efficiency of YAP and S100A7 was determined by western blot (Fig 6E and 6F). These data at least demonstrate that S100A7 plays the similar effect as YAP on HCC94 and FaDu cells proliferation, implying that S100A7 may act as a substitute for YAP in well differentiated cervical and glossopharyngeal SCC during activation of the Hippo pathway.

## Discussion

Cell behavior is not only governed by chemical signals but is also controlled by cell shape and cell density.[26] Indeed, our previous study proved that S100A7 was strongly induced in several SCC cells by suspension and dense culture.[11] In the present study, we demonstrate for the first time that Hippo-YAP pathway controls S100A7 expression in cervical and glossopharyngeal SCC mainly depending on the degree of cell differentiation. When the Hippo pathway is activated by either suspension and dense cultures or pharmacological perturbation of the actin cytoskeleton, a decrease in nuclear YAP results in S100A7 induction in HCC94 and FaDu cells (well differentiated SCC cells) while inactivation of the Hippo pathway suppresses S100A7 expression in these attached cells. Although the basic level of S100A7 expression is very low or undetectable in SiHa and H226 cells (poorly differentiated cells), we identify an unexpected phenomenon: S100A7 is hardly induced in these two cells even in the same condition as above, whereas the Hippo pathway is significantly activated. These data indicate that the Hippo-YAP pathway controlling S100A7 induction in cervical and pharyngeal SCC cells is determined by either differentiated status of SCC cells or some unknown intrinsic characters of cells, such as Lkb1 deficient. [27] More importantly, the positive correlation between S100A7 and YAP-S127 and the negative correlation between S100A7 with nuclear YAP were consistently observed in all of the well differentiated and more than half of moderately differentiated cervical and lingual SCC specimens but not in all of the poorly differentiated tissues.

Although several transcription factors, including ErbB4, Runx2, and TEAD, have been reported to interact with YAP, TEAD represent the major target transcription factors of YAP.[28–32] We further prove that the repressor function of nuclear YAP on S100A7 induction in well differentiated HCC94 and FaDu cells required TEAD1 but not the other members of this family. Our findings demonstrate that YAP-TEAD complex not only participated in gene activation but also gene repression. Indeed, a recent study has provided a direct evidence to support our opinion. They found that YAP/TAZ-TEAD function as transcriptional co-repressors via recruitment of the nucleosome remodeling and histone deacetylase (NuRD) complex to target gene, and this repressor function requires TEAD1/3/4 transcription.[33] Conversely, YAP-TAED acts as transcriptional co-activators via recruitment of the NcoA6 H3K4 methyltransferase complex and SWI/SNF chromatin-remodeling complex to target gene.[34,35] Therefore, we have reasons to believe that the repression of YAP-TEAD1 on S100A7 induction may be partially mediated by the recruitment of NuRD, although the precious mechanism is not elucidated in the present study. Collectively, our studies provide the first biological evidence that YAP functions as a transcriptional co-repressor to inhibit S100A7 expression via binding with TEAD1 in well differentiated cervical and pharyngeal SCC cells.

Both suspension and dense culture result in actin cytoskeleton remodeling, which promotes YAP phosphorylation and inhibit its transcriptional activity through activation of the Hippo pathway.[20,23] Similarly, we demonstrated that S100A7 expression and YAP phosphorylation were significantly increased in HCC94 and FaDu cells after pharmacological perturbation of the actin cytoskeleton. However, disruption of F-actin had no effect on S100A7 induction in poorly differentiated SiHa and H226 cells in spite of activation of the Hippo pathway. Although it is not clear whether the contractility mechanism per se or contractility just attains a specific cell shape and cytoskeleton organization to determine S100A7 expression in well differentiated SCC cells, F-actin remodeling really participates in control of S100A7 expression during the cells experiencing low mechanical stresses. Besides the actin cytoskeleton, the microtubule cytoskeleton is also reorganized during cell detachment and it also plays important roles in stimulating Lats1/2 kinase activity and YAP phosphorylation. Disruption of microtubule polymerization by nocodazole strongly blocked detachment-induced YAP phosphorylation but not affected attachment-induced YAP dephosphorylation.[20] In addition, the Hippo-YAP pathway is also regulated by G-protein coupled receptor signaling and protease activated receptor PAR.[36] Recently, several studies provide evidence that disruption of the E-cadherin-catenin complex at cell-cell junction also leads to activation of YAP.[37] Therefore, S100A7 induction in well differentiated cervical and pharyngeal SCC cells may also be regulated by several signaling pathways including chemical signals and tissue architecture as well as mechanical forces.

Although S100A7 expression and YAP activity displayed the negative correlation in well differentiated HCC94 and FaDu SCC cells and the clinical cervical and lingual SCC specimens, our data indeed support that both may perform the same or similar effects on cells proliferation and/or differentiation in well differentiated cervical and glossopharyngeal SCC. Several clues support our hypotheses. First, YAP plays a crucial role in organ size by promoting cell proliferation and inhibiting apoptosis.[16] Similarity, in the present study, we demonstrated that single depletion of YAP inhibited both HCC94 and FaDu cells proliferation. Second, our previous study demonstrated that single deletion of S100A7 inhibited HCC94 cells proliferation but promoted cell differentiation. However, the opposite results were obtained in FaDu cells by overexpression of S100A7.[11] Third, double silencing of YAP and S100A7 suppressed HCC94 cells proliferation more effective than single deletion of YAP and S100A7 (Fig 6C). As expected, the proliferation of FaDu cells was slightly influenced by combination of YAP-silenced and S100A7-overexpressed (Fig 6D). Fourth, S100A7 is responsible for cell detachment induced-anoikis resistance and tumorigenicity in human oral cancer cells.[20,38] Thus, we speculate that YAP and S100A7 might act as the compensatory function depending on cell microenvironment in well differentiated cervical and glossopharyngeal SCC. When these cells are detached or cultured in high density, the Hippo pathway is activated and nuclear YAP is attenuated, which leads to S100A7 induction in order to perform the similar effects as YAP, such as maintenance of cell survival and/or suppression of cell differentiation. With regard to the loss of YAP expression in some cervical and lingual SCC tissues, we consider that some other pathway may be responsible for cell proliferation in these cells but the mechanism is beyond the scope of this present study.

In summary, our findings prove a novel function and a critical role of the Hippo-YAP pathway in regulation of S100A7 expression in well differentiated cervical and glossopharyngeal SCC. Despite the fact that only four types of SCC cell lines and two kinds of SCC tissues are involved in the present study, this characteristic of S100A7 induction by the Hippo-YAP pathway may be the common features in SCCs. Therefore, this study not only demonstrates the complexity of SCCs but also provides potential opportunities for the development of new SCCs therapeutics.

The authors declare no potential conflicts of interest.

## Supporting Information

S1 Text The full description of materials and methods. (DOC)

S1 Table The sequences of siRNAs. (DOC)

S2 Table The primer sequences for qPCR. (DOC)

S1 Fig HCC94, FaDu and SiHa cells morphology in sparse and dense culture. (a) HCC94, FaDu and SiHa cells were seeded to obtain sparse and dense cells. Sparse: 7 500 cells/cm^2^; Dense: 75 000–100 000 cells/cm^2^. After two days, cells were visualized by AxioObserverD1. Scale bar, 200μm.
(TIF)

S2 Fig The transfection efficiency of two different specific LATS1 and MST1 siRNAs. (a-d) The silencing efficiency of LATS1 and MST1 siRNAs is detected by western blotting (Upper panel: a and b) and qPCR (Lower panel:c and d).
(TIF)

S3 Fig The Hippo pathway is responsible for S100A7 induction in well- and moderately differentiated SCC cells. (a-d) The mRNA levels of *CTGF*, *CYR61* and *S100A7* are analyzed by qRT-PCR. siLATS1+S48h (or D48h) indicates that cells are cultured in suspension (or dense) for 48 h after silencing of LATS1. siMST1+S48h (or D48h) indicates that cells are cultured in suspension (or dense) for 48 h after losing of MST1. H: HCC94 cells; F: FaDu cells. Error bar, SD of three different experiments. *P<0.05, **P<0.01; t-test
(TIF)

S4 Fig F-actin disruption enhances S100A7 mRNA level via activation of the Hippo pathway in well- and moderately differentiated SCC cells. (a, b) The expression of *CTGF*, *CYR61* and *S100A7* in LatB (a) or Cyto D (b) treated HCC94 and FaDu cells is detected by qRT-PCR. Error bar, SD of three different experiments. *P<0.*05*, **P<0.01; *t-test*. (c) C3 (1 μg/ml) was added to HCC94 (c, left) and FaDu (c, right) cells with serum-free growth medium for 4 h prior to harvesting for qRT-PCR. H: HCC94 cells; F: FaDu cells. Error bar, SD of three different experiments. *P<0.*05*, **P<0.01; *t-test*.
(TIF)

S5 Fig Depletion of TEAD2/3/4 can not induce S100A7 mRNA level in HCC94 cells. (a-c) RT-PCR analyses for S100A7 expression after TEAD2 (a), TEAD3 (b) and TEAD4 (c) silencing in normal attached HCC94 cells. β-actin was used as a loading control.
(TIF)

S6 Fig All the immunohistochemistry images showing S100A7, pYAP-S127 and total YAP staining in 25 cases of well differentiated cervical SCC tissues. (a) Sample 1–17 are S100A7+/pYAP-S127+/YAP+ tissues.Sample 18–22 are S100A7+/pYAP-S127^-^/YAP+ tissues. Sample 23–25 are S100A7+/pYAP-S127^-^/YAP^-^ tissues.
(TIF)

## References

[1] Kulski JK, Lim CP, Dunn DS, Bellgard M. Phylogenetic Analysis of the S100A7 (Psoriasin) Gene Duplications Within the Region of the S100 Gene Cluster on Human Chromosome 1q21. J Mol Evol. 2003; 56: 397–406. 10.1007/s00239-002-2410-5 12664160

[2] Peder M, Hanne HR, Henrik L, Bent H, Kurt D, Eydfinnur O, et al Molecular cloning, occurrence, and expression of a novel partially secreted protein "psoriasin" that is highly up-regulated in psoriatic skin. J Invest Dermatol. 1991; 97: 701–12. 1940442 10.1111/1523-1747.ep12484041

[3] Alowami S, Qing G, Emberley E, Snell L, Watson PH. Psoriasin (S100A7) expression is altered during skin tumorigenesis. BMC Dermatol. 2003; 3: 1–7. 10.1186/1471-5945-3-1 12600274 PMC151671

[4] Nasser MW, Qamri Z, Deol YS, Ravi J, Powell CA, Trikha P, et al S100A7 enhances mammary tumorigenesis through upregulation of inflammatory pathways. Cancer Res. 2012; 72: 604–615. 10.1158/0008-5472.CAN-11-0669 22158945 PMC3271140

[5] Celis JE, Rasmussen HH, Vorum H, Madsen P, Honoré B, Wolf H, et al Bladder squamous cell carcinomas express psoriasin and externalize it to the urine. J Urol. 1996; 155: 2105–2112. 8618345

[6] Mandal S, Curtis L, Pind M, Murphy LC, Watson PH. S100A7 (psoriasin) influences immune response genes in human breast cancer. Exp Cell Res. 2007; 313: 3016–3025. 10.1016/j.yexcr.2007.03.020 17560571

[7] Moubayed N, Weichenthal M, Harder J, Wandel E, Sticherling M, Gläser R. Psoriasin (S100A7) is significantly up-regulated in human epithelial skin tumours. J Cancer Res Clin Oncol. 2007; 133: 253–261. 10.1007/s00432-006-0164-y 17136347 PMC12160872

[8] Kesting MR, Sudhoff H, Hasler RJ, Nieberler M, Pautke C, Wolff KD, et al Psoriasin (S100A7) up-regulation in oral squamous cell carcinoma and its relation to clinicopathologic features. Oral Oncol. 2009; 45: 731–736. 10.1016/j.oraloncology.2008.11.012 19147391

[9] Tripathi SC, Matta A, Kaur J, Grigull J, Chauhan SS, Thakar A, et al Nuclear S100A7 Is Associated with Poor Prognosis in Head and Neck Cancer. PLoS One. 2010; 5: 1–10. 10.1371/journal.pone.0011939 PMC2914786 20689826

[10] Barbieri MR, Andrade CD, Silva WA Jr, Marques AA, Leopoldino AM, Montes MB, et al Expression of human protein S100A7 (psoriasin), preparation of antibody and application to human larynx squamous cell carcinoma. BMC Res Notes. 2011; 4: 1–8. 22082027 10.1186/1756-0500-4-494 PMC3278597

[11] Qi Z, Li T, Kong F, Li YG, Wang R, Wang JH, et al The Characteristics and Function of S100A7 Induction in Squamous Cell Carcinoma: Heterogeneity, Promotion of Cell Proliferation and Suppression of Differentiation. PLoS One. 2015; 10: 1–16. 10.1371/journal.pone.0128887 PMC4460013 26053695

[12] Li T, Qi Z, Kong F, Li YG, Wang R, Zhang WQ, et al S100A7 acts as a dual regulator in promoting proliferation and suppressing squamous differentiation through GATA-3/caspase-14 pathway in A431 cells. Exp Dermatol. 2015; 24: 342–348. 10.1111/exd.12645 25651379

[13] Li YG, Kong F, Wang JH, Hu EZ, Wang R, Liu J, et al S100A7 induction is repressed by YAP via the Hippo pathway in A431 cells. Oncotarget. 2016. 10.18632/oncotarget.9477 PMC5122377 27203549

[14] Meng Z, Moroishi T, Mottier-Pavie V, Plouffe SW, Hansen CG, Hong AW, et al MAP4K family kinases act in parallel to MST1/2 to activate LATS1/2 in the Hippo pathway. Nature Communications. 2015; 6:1–13. 10.1038/ncomms9357 PMC4600732 26437443

[15] Chan EH, Nousiainen M, Chalamalasetty RB, Schäfer A, Nigg EA, Silljé HH. The Ste20-like kinase Mst2 activates the human large tumor suppressor kinase Lats1. Oncogene. 2005; 24: 2076–2086. 10.1038/sj.onc.1208445 15688006

[16] Zhao B, Wei X, Li W, Udan RS, Yang Q, Kim J, et al Inactivation of YAP oncoprotein by the Hippo pathway is involved in cell contact inhibition and tissue growth control. Genes Dev. 2007; 21: 2747–2761. 10.1101/gad.1602907 17974916 PMC2045129

[17] Callus BA, Verhagen AM, Vaux DL. Association of mammalian sterile twenty kinases, Mst1 and Mst2, with hSalvador via C-terminal coiled-coil domains, leads to its stabilization and phosphorylation. FEBS J. 2006; 273: 4264–4276. 10.1111/j.1742-4658.2006.05427.x 16930133

[18] Hao Y, Chun A, Cheung K, Rashidi B, Yang X. Tumor Suppressor LATS1 Is a Negative Regulator of Oncogene YAP. J Biol Chem. 2008; 283: 5496–5509. 10.1074/jbc.M709037200 18158288

[19] Schlegelmilch K, Mohseni M, Kirak O, Pruszak J, Rodriguez JR, Zhou D, et al Yap1 acts downstream of α-catenin to control epidermal proliferation. Cell. 2011; 144: 782–795. 10.1016/j.cell.2011.02.031 21376238 PMC3237196

[20] Zhao B, Li L, Wang L, Wang CY, Yu J, Guan KL. Cell detachment activates the Hippo pathway via cytoskeleton reorganization to induce anoikis. Genes Dev. 2013; 26: 54–68. 10.1101/gad.173435.111 PMC3258966 22215811

[21] Wada K, Itoga K, Okano T, Yonemura S, Sasaki H. Hippo pathway regulation by cell morphology and stress fibers. Development. 2011; 138: 3907–3914. 10.1242/dev.070987 21831922

[22] Martinsson H, Yhr M, Enerback C. Expression patterns of S100A7 (psoriasin) and S100A9 (calgranulin-B) in keratinocyte differentiation. Exp Dermatol. 2005; 14: 161–168. 10.1111/j.0906-6705.2005.00239.x 15740587

[23] Aragona M, Panciera T, Manfrin A, Giulitti S, Michielin F, Elvassore N, et al A Mechanical Checkpoint Controls Multicellular Growth through YAP/TAZ Regulation by Actin-Processing Factors. Cell. 2013; 154: 1047–1059. 10.1016/j.cell.2013.07.042 23954413

[24] Zhang K, Qi HX, Hu ZM, Chang YN, Shi ZM, Han XH, et al YAP and TAZ Take Center Stage in Cancer. Biochemistry. 2015; 54: 6555–6566. 10.1021/acs.biochem.5b01014 26465056

[25] Kim M, Kim T, Johnson RL, Lim DS. Transcriptional Co-repressor Function of the Hippo Pathway Transducers YAP and TAZ. Cell Rep. 2015; 11: 270–282. 10.1016/j.celrep.2015.03.015 25843714

[26] Halder G, Dupont S, Piccolo S. Transduction of mechanical and cytoskeletal cues by YAP and TAZ. Nat Rev Mol Cell Biol. 2012; 13: 591–600. 10.1038/nrm3416 22895435

[27] Han X, Li F, Fang Z, Gao Y, Li F, Fang R, et al Transdifferentiation of lung adenocarcinoma in mice with *Lkb1* deficiency to squamous cell carcinoma. Nat Commun. 2014; 5: 3261 10.1038/ncomms4261 24531128 PMC3929783

[28] Vassilev A, Kaneko KJ, Shu H, Zhao Y, DePamphilis ML. TEAD/TEF transcription factors utilize the activation domain of YAP65, a Src/Yes-associated protein localized in the cytoplasm. Genes Dev. 2001; 15: 1229–1241. 10.1101/gad.888601 11358867 PMC313800

[29] Zhao B, Ye X, Yu J, Li L, Li W, Li S, Yu J, et al TEAD mediates YAP-dependent gene induction and growth control. Genes Dev. 2008; 22: 1962–1971. 10.1101/gad.1664408 18579750 PMC2492741

[30] Yagi R, Chen LF, Shigesada K, Murakami Y, Ito Y. A WW domain-containing yes-associated protein(YAP) is a novel transcriptional co-activator. EMBO J. 1999; 18: 2551–2562. 10.1093/emboj/18.9.2551 10228168 PMC1171336

[31] Basu S, Totty NF, Irwin MS, Sudol M, Downward J. Akt phosphorylates the Yes-associated protein, YAP, to induce interaction with 14–3–3 and attenuation of p73-mediated apoptosis. Mol Cell. 2003; 11: 11–23. 12535517 10.1016/s1097-2765(02)00776-1

[32] Komuro A, Nagai M, Navin NE, Sudol M. WW Domain-containing Protein YAP Associates with ErbB-4 and Acts as a Co-transcriptional Activator for the Carboxyl-terminal Fragment of ErbB-4 That Translocates to the Nucleus. J Biol Chem. 2003; 278: 33334–33341. 10.1074/jbc.M305597200 12807903

[33] Mullen AC. Hippo Tips the TGF-β Scale in Favor of Pluripotency. Cell Stem Cell. 2014; 14: 6–8. 10.1016/j.stem.2013.12.009 24388171 PMC3941189

[34] Oh H, Slattery M, Ma L, White KP, Mann RS, Irvine KD. Yorkie promotes transcription by recruiting a Histone methyltransferase complex. Cell Rep. 2014; 8: 449–459. 10.1016/j.celrep.2014.06.017 25017066 PMC4152371

[35] Zhu Y, Li D, Wang Y, Pei C, Liu S, Zhang L, et al Brahma regulates the Hippo pathway activity through forming complex with Yki-Sd and regulating the transcription of Crumbs. Cell Signal. 2015; 27: 606–13. 10.1016/j.cellsig.2014.12.002 25496831

[36] Mo JS, Yu FX, Gong R, Brown JH, Guan KL. Regulation of the Hippo–YAP pathway by protease-activated receptors (PARs). Genes Dev. 2012; 26: 2138–2143. 10.1101/gad.197582.112 22972936 PMC3465735

[37] Kim NG, Koh E, Chen X, Gumbiner BM. E-cadherin mediates contact inhibition of proliferation through Hippo signaling-pathway components. Proc Natl Acad Sci U S A. 2011; 108: 11930–11935. 10.1073/pnas.1103345108 21730131 PMC3141988

[38] Dey KK, Sarkar S, Pal I, Das S, Dey G, Bharti R, et al Mechanistic attributes of S100A7 (psoriasin) in resistance of anoikis resulting tumor progression in squamous cell carcinoma of the oral cavity. Cancer Cell Int. 2015; 15: 1–11. 26225121 10.1186/s12935-015-0226-9 PMC4518584

