# Supplementary material for: The Characteristic of S100A7 Induction by the Hippo-YAP Pathway in Cervical and Glossopharyngeal Squamous Cell Carcinoma
Source: PLoS One. 2016 Dec 1;11(12):e0167080. doi: 10.1371/journal.pone.0167080 (PMC5132200; doi:10.1371/journal.pone.0167080)
Supplement: S1 Text — (DOC) [file pone.0167080.s001.doc]

**S1 Text**

**Plasmids and Reagents**

The pcDNA4-His-YAP WT; S127A and pCMV14-Flag-YAP WT; S94A vectors were kindly provided by Dr. Zhang (Mayo Clinic College of Medicine, USA). For pCMV14-Flag-LATS1, the LATS1 (NCBI Gene ID: 9113) cDNA fragment was amplified using 5’-CGGGGTACCATGAAGAGGAGTGAAAAG-3’and 5’-GCTCTAGAAACATATACTAGATCGCGATTT-3’, and then was cloned into the mammalian expression vector pCMV14 (Invitrogen, Carlsbad, CA, USA) using *KpnI* and *XbaI* restriction enzymes (Takara, Dalian, China). Latrunculin B (L5288) and Cytochalasin D (C8273) were purchased from Sigma (Sigma-Aldrich Ltd, Dorset, UK). Botulinum toxin C3 (CT04) was purchased from Cytoskeleton (Denver, USA).

**Western blot**

Western blotting analysis was performed as previously described.[1] The following antibodies were used: MST1 (1/1000; Cell Signaling Technology, 3682, Boston, USA); LATS1 (1/1000; Cell Signaling Technology, 3477); pLATS1-T1079 (1/1000; Cell Signaling Technology, 8654); S100A7 (1/1000; Abcam, ab13680, Cambridge, UK); YAP (1/1000; Cell Signaling Technology, 4912); pYAP-S127 (1/1000; Cell Signaling Technology, 13008S); anti-Flag tag (CWBIO, CW0287A, Beijing, China); anti-His tag (MBL, D291-3, NAGOYA, JAPAN). GAPDH (ZSGB-BIO, TA-08, Beijing, China) and β-actin (ZSGB-BIO, TA-09) were used as loading controls.

**Immunofluorescence staining**

For suspended cells, cells were cultured in suspension for 24 hours and reattached to a slide for 12 h. Cells were fixed with 3.4% paraformaldehyde for 20 min and then permeabilized with 0.5% PBS-Triton X-100 (Dingguo, Beijing, China). After blocking in 3% PBS-BSA for 30 min, slides were incubated with anti-S100A7 (1/200) and anti-YAP (1/500); antibodies diluted in 1% BSA for 1 h at 37℃. After washing with PBS, slides were incubated with goat anti-mouse TRTIC (tetramethyl thodamine isothiocyanate) 555- (ZSGB-BIO, ZF-0316) or goat anti-Rabbit FITC (fluorescein isothicocyanate) 488- (ZSGB-BIO, ZF-0312) conjugated secondary antibodies for 1 h at 37℃. The nuclei were stained using DAPI (4’, 6-diamidino-2-phenylindole). The targeted proteins were detected using confocal microscopy (ZEISS LSM700, Oberkochen, Germany) and a ZEISS LSM700 laser-scanning confocal microscope image system. Nonspecific IgG was used as a negative control.

**Immunohistochemistry**
Immunohistochemistry was performed as described in our previous study.[1] Anti-S100A7 (1/200), anti-pYAP-S127 (1/200) and anti-YAP (1:100) were separately incubated with the specimens. The goat anti-Rabbit/Mouse secondary antibody was purchased from MAIXINBIO (KIT-5010, Fujian, China). S100A7, pYAP-S127 and YAP expression was detected using a light microscope (ZEISS ImagerA1).

**MTT assay**

MTT assay performed as described in our previous study.[2]

**Statistical analysis**

Statistical analysis was performed using GraphPad Prism software. The statistical significance was evaluated using Student’s *t*-test (2-tailed) to compare two groups of data. The asterisks indicate significant differences between the experimental groups and corresponding control condition. Differences were considered statistically significant at a *P*-value of less than 0.05. *P-*values <0.05, <0.01 are indicated with one and two asterisks, respectively.

1. Zhang H, Zhao Q, Chen Y, Wang Y, Gao S, Mao Y et al. Selective expression of S100A7 in lung squamous cell carcinomas and large cell carcinomas but not in adenocarcinomas and small cell carcinomas. [Thorax](http://www.ncbi.nlm.nih.gov/pubmed/?term=Selective+expression+of+S100A7+in+lung+squamous+cell+carcinomas+and+large+cell+carcinomas+but+not+in+adenocarcinomas+and+small+cell+carcinomas#%23). 2008; 63: 352–359.

# 2. Qian Z, Li M, Wang R, Xian Q, Wang J, Li M et al. Knockdown of CABYR-a/b increases chemosensitivity of human non-small cell lung cancer cells through inactivation of Akt. [Mol Cancer Res](http://www.ncbi.nlm.nih.gov/pubmed/24362251).2014; 12: 335-47
