# Supplementary material for: The Characteristic of S100A7 Induction by the Hippo-YAP Pathway in Cervical and Glossopharyngeal Squamous Cell Carcinoma
Source: PLoS One. 2016 Dec 1;11(12):e0167080. doi: 10.1371/journal.pone.0167080 (PMC5132200; doi:10.1371/journal.pone.0167080)
Supplement: S1 Table — (DOC) [file pone.0167080.s002.doc]

**S1 Table**

| Target Gene | siRNA sequences |
| --- | --- |
| YAP-sense | 5' GGUGAUACUAUCAACCAAATT 3' |
| YAP-antisense | 5' UUUGGUUGAUAGUAUCACCTT 3' |
| LATS1#A-sense | 5' GAGCUGGAAAGGUUCUAAATT 3' |
| LATS1#A-antisense | 5' UUUAGAACCUUUCCAGCUCTT 3' |
| LATS1#B-sense | 5' GCAGCGUCUACAUCGUAAATT 3' |
| LATS1#B-antisense | 5' UUUACGAUGUAGACGCUGCTT 3' |
| MST1#A-sense | 5' GGACCUGCAUCAUGAACAATT 3' |
| MST1#A-antisense | 5' UUGUUCAUGAUGCAGGUCCTT 3' |
| MST1#B-sense | 5' GCUUCUCCUCCUGCCAUAUTT 3' |
| MST1#B-antisense | 5' AUAUGGCAGGAGGAGAAGCTT 3' |
| TEAD1#A-sense | 5' GCCACUGCCAUUCAUAACATT 3' |
| TEAD1#A-antisense | 5' UGUUAUGAAUGGCAGUGGCTT 3' |
| TEAD1#B-sense | 5' AUGGCCGAUUUGUAUACCGAATT 3' |
| TEAD1#B-antisense | 5' UUCGGUAUACAAAUCGGCCAUTT 3' |
| TEAD2#A-sense | 5’CGGCAGAUCUACGACAAAUTT 3’ |
| TEAD2#A-antisense | 5’ AUUUGUCGUAGAUCUGCCGTT3’ |
| TEAD2#B-sense | 5’GCCAGAUGCAGUUGAUUCUTT3’ |
| TEAD2#B-antisense | 5’AGAAUCAACUGCAUCUGGCTT3’ |
| TEAD3#A-sense | 5’CCAGUGUCCUGCAGAACAATT3’ |
| TEAD3#A-antisense | 5’UUGUUCUGCAGGACACUGGTT3’ |
| TEAD3#B-sense | 5’GACCCUCUCAGGACAUCAATT3’ |
| TEAD3#B-antisense | 5’UUGAUGUCCUGAGAGGGUCTT3’ |
| TEAD4#A-sense | 5’CCGCCAAAUCUAUGACAAATT3’ |
| TEAD4#A-antisense | 5’UUUGUCAUAGAUUUGGCGGTT3’ |
| TEAD4#B-sense | 5’CCACGAAGGUCUGCUCUUUTT3’ |
| TEAD4#B-antisense | 5’AAAGAGCAGACCUUCGUGGTT3’ |
