# Supplementary material for: The Characteristic of S100A7 Induction by the Hippo-YAP Pathway in Cervical and Glossopharyngeal Squamous Cell Carcinoma
Source: PLoS One. 2016 Dec 1;11(12):e0167080. doi: 10.1371/journal.pone.0167080 (PMC5132200; doi:10.1371/journal.pone.0167080)
Supplement: S2 Table — (DOC) [file pone.0167080.s003.doc]

**S2 Table**

| Gene | Primers sequences |
| --- | --- |
| S100A7-sense | 5' CTTCCCCAACTTCCTTAGTG 3' |
| S100A7-antisense | 5' GTAGTCTGTGGCTATGTCTC 3' |
| CYR61-sense | 5' GCTGCGAGGAGTGGGTCTGT 3' |
| CYR61-antisense | 5' GGGTTGTATAGGATGCGAGGCT 3' |
| CTGF-sense | 5' GCATCCGTACTCCCAAAATCTC 3' |
| CTGF-antisense | 5' CAGGGCACTTGAACTCCACC 3' |
| GAPDH-sense | 5' GAGTCAACGGATTTGGTCGT 3' |
| GAPDH-antisense | 5' GACAAGCTTCCCGTTCTCAG 3' |
| YAP-sense | 5’CCTCTATTTTGCTCTTCCTTGTCC3’ |
| YAP-antisense | 5’CCATCATCCAAACAGGCTCAC3’ |
| TEAD1-sense | 5’TCGAGCAGCAGCGAGACCCAGACTC3’ |
| TEAD1-antisense | 5’TTACGAGGAAGAAGGCATTTTGAGG3’ |
| TEAD2-sense | 5’ TGCCTTCTTCCTGGTCAAGTTCTG 3’ |
| TEAD2-antisense | 5’ CTCATACTGGCTGCTCACTCCGT 3’ |
| TEAD3-sense | 5’ TCCTGTCAGACGAGGGCAAGATG 3’ |
| TEAD3-antisense | 5’ CTTCCGAGCTAGAACCTGTATGTG 3’ |
| TEAD4-sense | 5’ TTGAGCAGAGTTTCCAGGAGGCC 3’ |
| TEAD4-antisense | 5’ CAATCAGCTCGTTCCGACCATACA 3’ |
