# Supplementary figures and images for: The Characteristic of S100A7 Induction by the Hippo-YAP Pathway in Cervical and Glossopharyngeal Squamous Cell Carcinoma
Source: PLoS One. 2016 Dec 1;11(12):e0167080. doi: 10.1371/journal.pone.0167080 (PMC5132200; doi:10.1371/journal.pone.0167080)

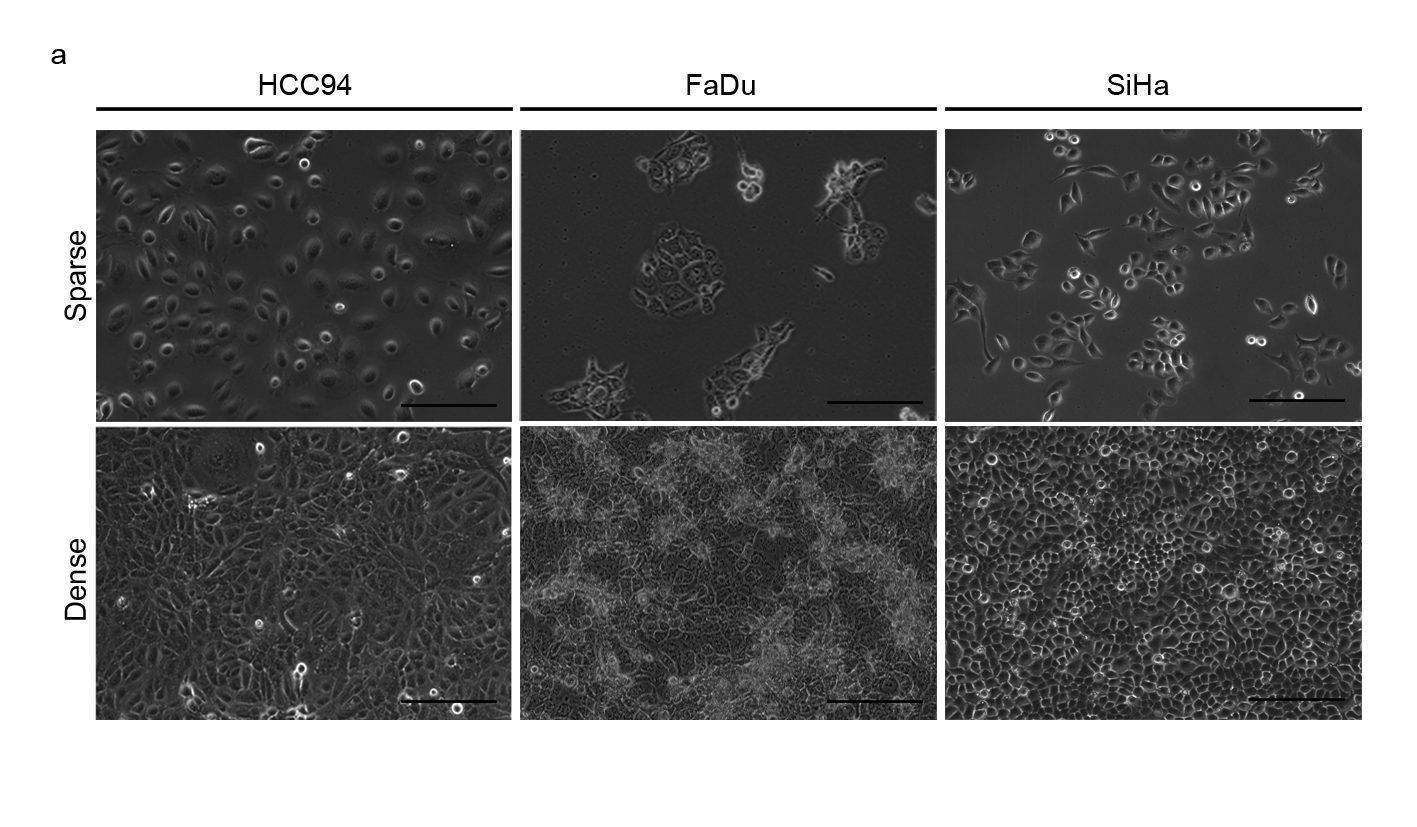

Supplement: S1 Fig — (a) HCC94, FaDu and SiHa cells were seeded to obtain sparse and dense cells. Sparse: 7 500 cells/cm2; Dense: 75 000–100 000 cells/cm2. After two days, cells were visualized by AxioObserverD1. Scale bar, 200μm. (TIF) [file pone.0167080.s004.tif]

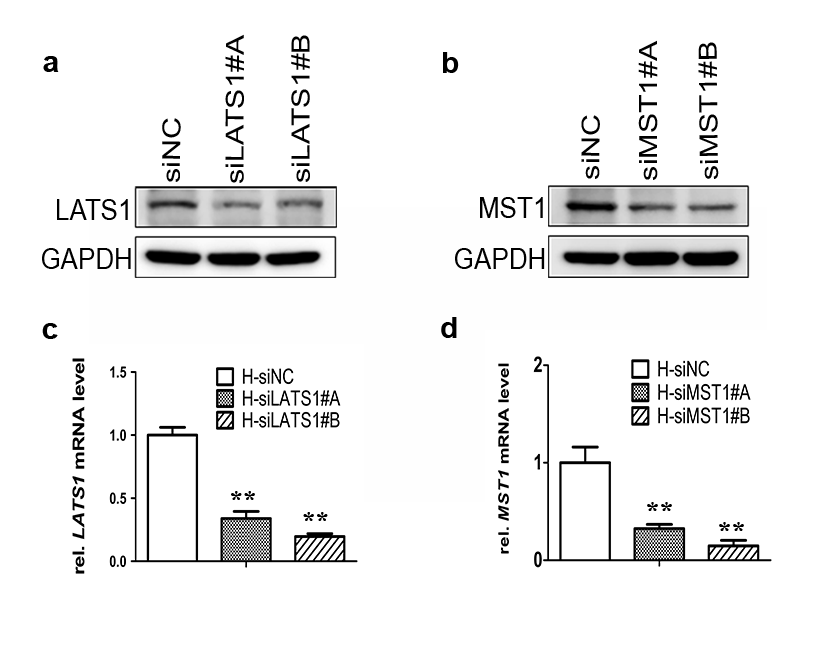

Supplement: S2 Fig — (a-d) The silencing efficiency of LATS1 and MST1 siRNAs is detected by western blotting (Upper panel: a and b) and qPCR (Lower panel:c and d). (TIF) [file pone.0167080.s005.tif]

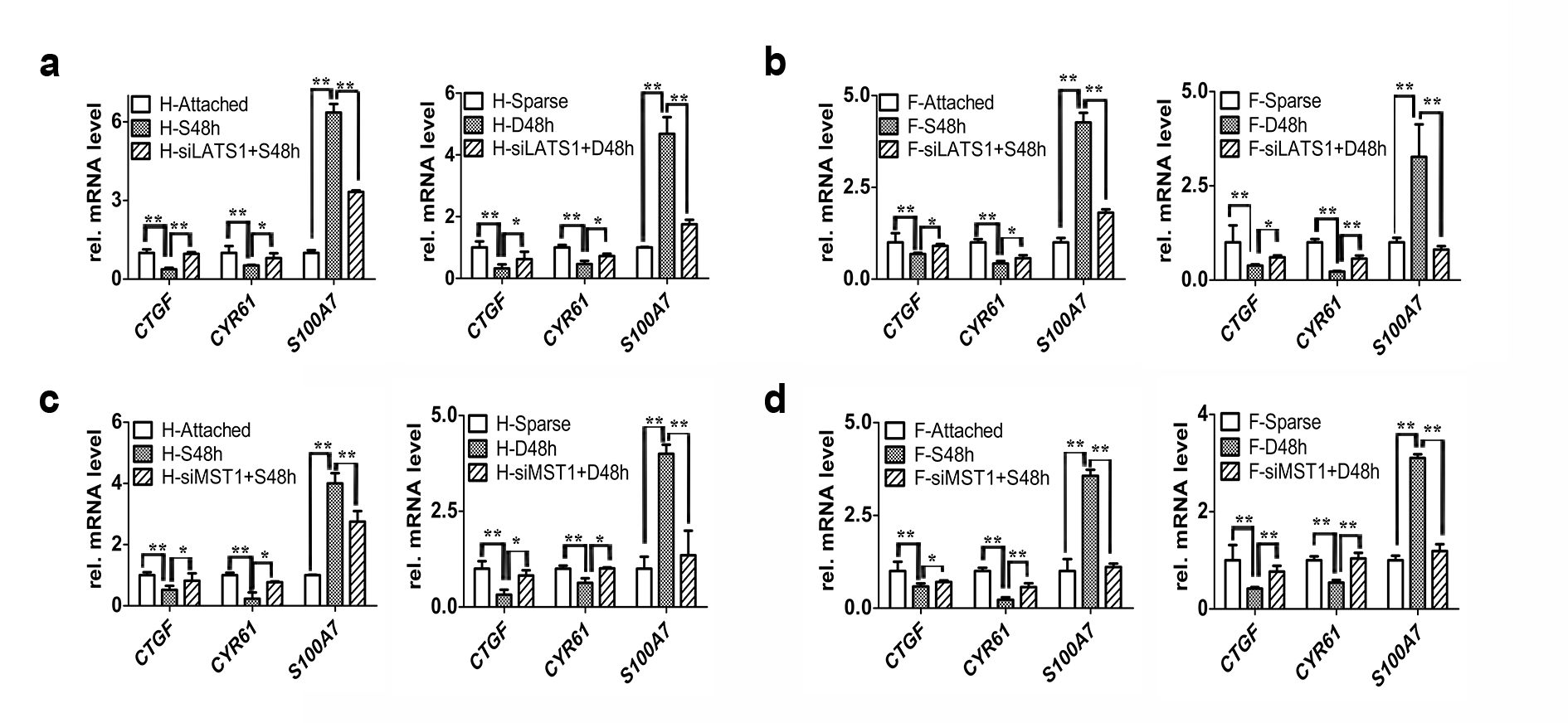

Supplement: S3 Fig — (a-d) The mRNA levels of CTGF, CYR61 and S100A7 are analyzed by qRT-PCR. siLATS1+S48h (or D48h) indicates that cells are cultured in suspension (or dense) for 48 h after silencing of LATS1. siMST1+S48h (or D48h) indicates that cells are cultured in suspension (or dense) for 48 h after losing of MST1. H: HCC94 cells; F: FaDu cells. Error bar, SD of three different experiments. *P<0.05, **P<0.01; t-test (TIF) [file pone.0167080.s006.tif]

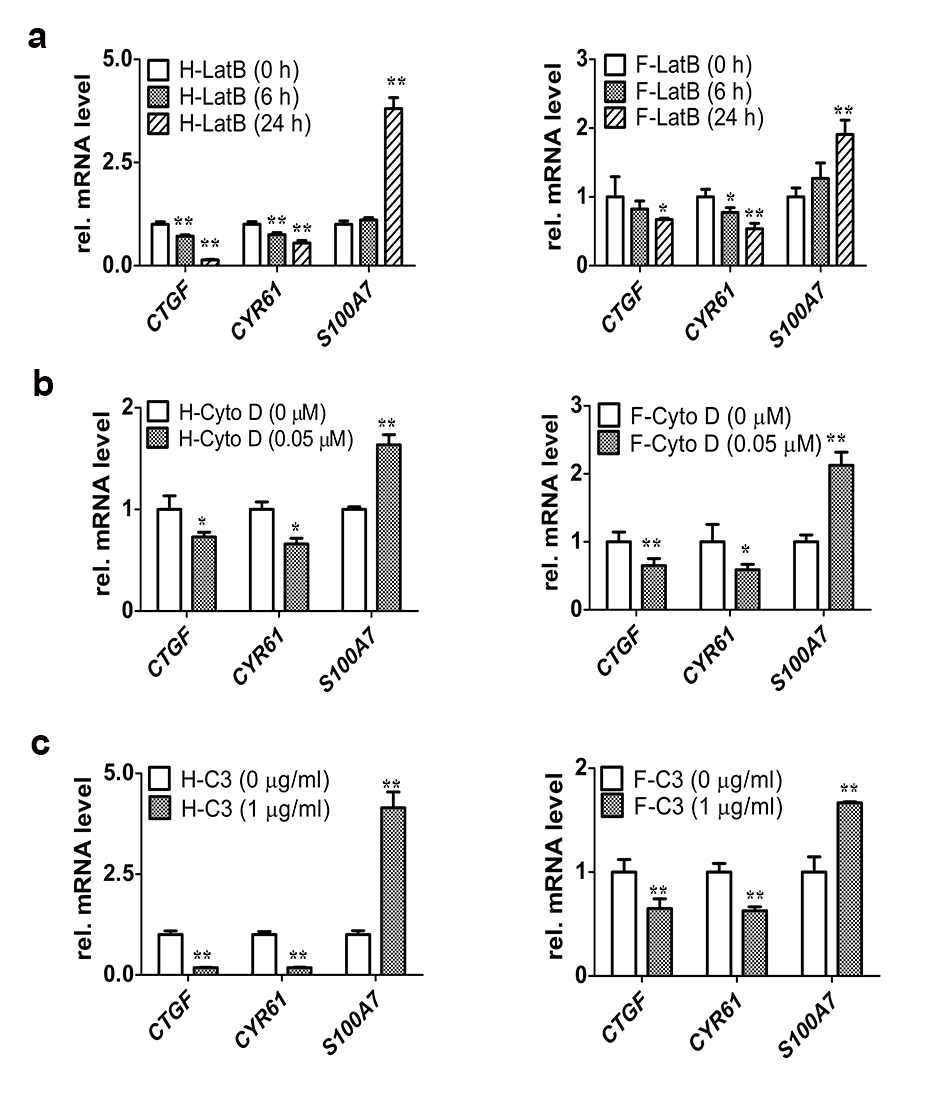

Supplement: S4 Fig — (a, b) The expression of CTGF, CYR61 and S100A7 in LatB (a) or Cyto D (b) treated HCC94 and FaDu cells is detected by qRT-PCR. Error bar, SD of three different experiments. *P<0.05, **P<0.01; t-test. (c) C3 (1 μg/ml) was added to HCC94 (c, left) and FaDu (c, right) cells with serum-free growth medium for 4 h prior to harvesting for qRT-PCR. H: HCC94 cells; F: FaDu cells. Error bar, SD of three different experiments. *P<0.05, **P<0.01; t-test. (TIF) [file pone.0167080.s007.tif]

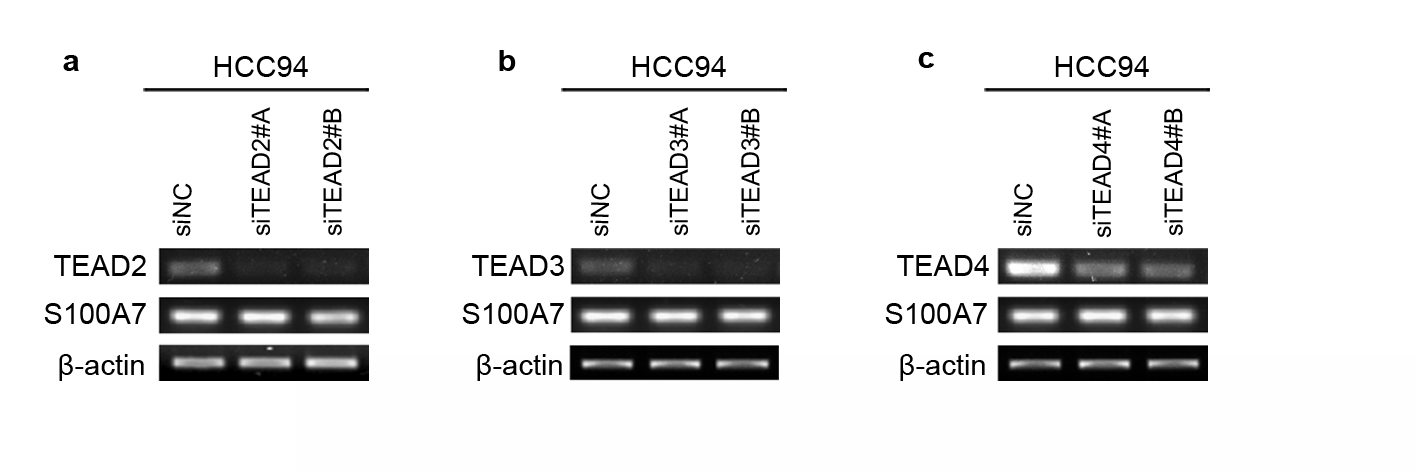

Supplement: S5 Fig — (a-c) RT-PCR analyses for S100A7 expression after TEAD2 (a), TEAD3 (b) and TEAD4 (c) silencing in normal attached HCC94 cells. β-actin was used as a loading control. (TIF) [file pone.0167080.s008.tif]

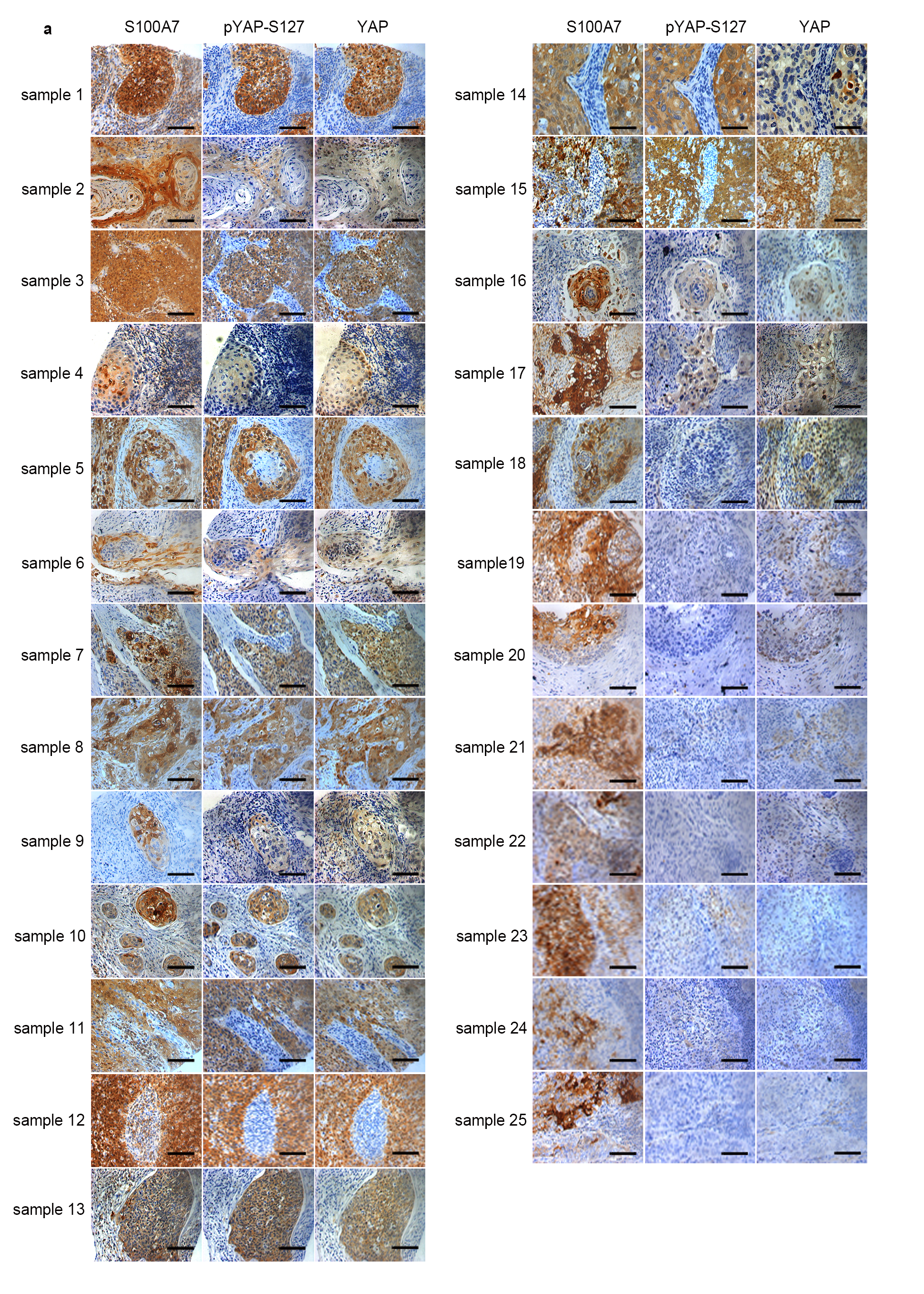

Supplement: S6 Fig — (a) Sample 1–17 are S100A7+/pYAP-S127+/YAP+ tissues.Sample 18–22 are S100A7+/pYAP-S127-/YAP+ tissues. Sample 23–25 are S100A7+/pYAP-S127-/YAP- tissues. (TIF) [file pone.0167080.s009.tif]
